# Supplementary material for: Molecular molds for regularizing Kondo states at atom/metal interfaces
Source: Nat Commun. 2020 May 22;11:2566. doi: 10.1038/s41467-020-16402-6 (PMC7244723; doi:10.1038/s41467-020-16402-6)
Supplement: Supplementary file 1 — Supplementary Information [file 41467_2020_16402_MOESM1_ESM.pdf]

Supplementary Information for

Molecular Molds for Regularizing Kondo States at Atom/Metal Interfaces

Li *et al.*

Contents

Supplementary Note 1: Topographic images and Kondo maps of the K_n complexes on Au(111) surface 3
Supplementary Note 2: The dI/dV spectra of K_n complexes measured at different tip positions 5
Supplementary Note 3: Manipulation of a K_1 complex on the Au(111) surface 6
Supplementary Note 4: Experimental determination of Kondo temperature and the influence of modulation voltage 7
Supplementary Note 5: Geometrical and electronic structures of the CoPc molecular mold 8
Supplementary Note 6: Geometrical and electronic structures of the K_n/Au(111) composites 9
Supplementary Note 7: Influence of adsorption site on the local spin state of captured Co adatom 12
Supplementary Note 8: Influence of surrounding environment on the local spin state of Co adatom 13
Supplementary Note 9: Parameters of Anderson impurity models for the K_n/Au(111) composites 15
Supplementary Note 10: Theoretical determination of Kondo temperatures of K_n/Au(111) composites 17
Supplementary Note 11: Binding energy of Co adatoms in the K_n/Au(111) composites 20
Supplementary Note 12: Experimental construction and measurement of K_n(CuPc) and K_n(H_2Pc) complexes 21
Supplementary Note 13: Electronic structures of $K_{2'}$(CuPc) and $K_{2'}$(H_2Pc) complexes 22
Supplementary Note 14: Existence/Absence of superexchange channel in the K_n/Au(111) composites 23
Supplementary Note 15: Analysis on the superexchange interaction in $K_{2'}$/Au(111) and K_4/Au(111) composites 26

Supplementary References 29

Supplementary Note 1: Topographic images and Kondo maps of the K_n complexes on Au(111) surface

Supplementary Figure 1(A) and (B) depict the topographic image and the differential conductance (dI/dV) map of a K_1 complex adsorbed on the Au(111) surface, respectively. In the former picture, the isoindole unit hosting a Co adatom is more protruded in comparison with those isoindole units without a captured Co adatom, and the center of the protrusion reflects the actual adsorption site of the captured Co adatom. However, in the simultaneously acquired dI/dV map at the condition of -15 mV and 2 nA, an area with lower value appears. The corresponding dI/dV curves are obtained by placing the STM tip at the positions marked by the white points, as shown in Supplementary Figure 1(C). The Kondo cloud centered at a captured Co adatom has a crescent shape distribution at the outer region of an isoindole unit.

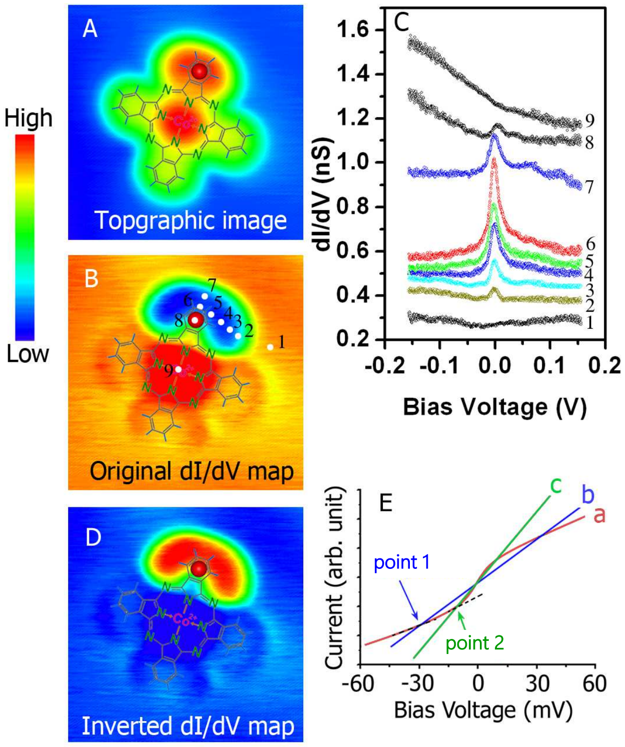

Supplementary Figure 1: (A) Topographic image and (B) simultaneously measured dI/dV map for a K_1 complex on the Au(111) surface, with the superposed molecular structure. (C) dI/dV curves measured at the marked white points in (B). The lines are shifted for clarity. (D) Inverted dI/dV map for the same K_1 complex, where the red area denotes the distribution of the Kondo cloud (Kondo map). (E) Illustration of I-V curves at different bias voltages (say, at point 1 or point 2). It displays the different slope of the curves measured at positions inside (curve a) and outside (curve b or curve c) the Kondo cloud. The contrast in dI/dV map is determined by the distribution in the slopes of different I-V curve at a given bias voltage. For a maximal contrast, the Kondo map is acquired at a bias voltage of -15 mV between points 1 and 2. Image size: $2.5\times2.5\,\mathrm{nm}^2$ for (A), (B), and (D).

As illustrated in Supplementary Figure 1(E), the curve a denotes the typical I-V behavior around a captured Co adatom, while the curve b (or c) denotes other sites far from the captured Co adatom. When we took dI/dV map at lower bias voltages, say at point 1 (or point 2), the slope of the curve a around the point 1 and point 2 is much smaller than that of curve b (or c), hence the dI/dV map gives lower value around the captured Co adatom than the other site far from it. For a maximal contrast, the dI/dV map in Supplementary Figure 1(B) is acquired at a bias voltage of -15 mV between the point 1 and point 2. The lower value area reflects the distribution of the Kondo cloud. The inverted dI/dV map in Supplementary Figure 1(D) thus gives a more direct view of the Kondo map, which is presented in the main text.

Supplementary Note 2: The dI/dV spectra of K_n complexes measured at different tip positions

For a K_n complex hosting two or more Co adatoms, the dI/dV spectra measured by placing the STM tip on top of different captured Co adatoms almost overlap each other; see Supplementary Figure 2. The central Co ion in any K_n complex is clearly Kondo inactive. In general, the spectral line shape of a K_n ($n \geq 2$) much resembles that of a K_1, but the zero-bias Kondo peak becomes broader as n increases from 1 to 4. Intrinsically, the Kondo peak in the dI/dV spectrum of a $K_{2'}$ complex is much wider than that of a K_2 or a K_3.

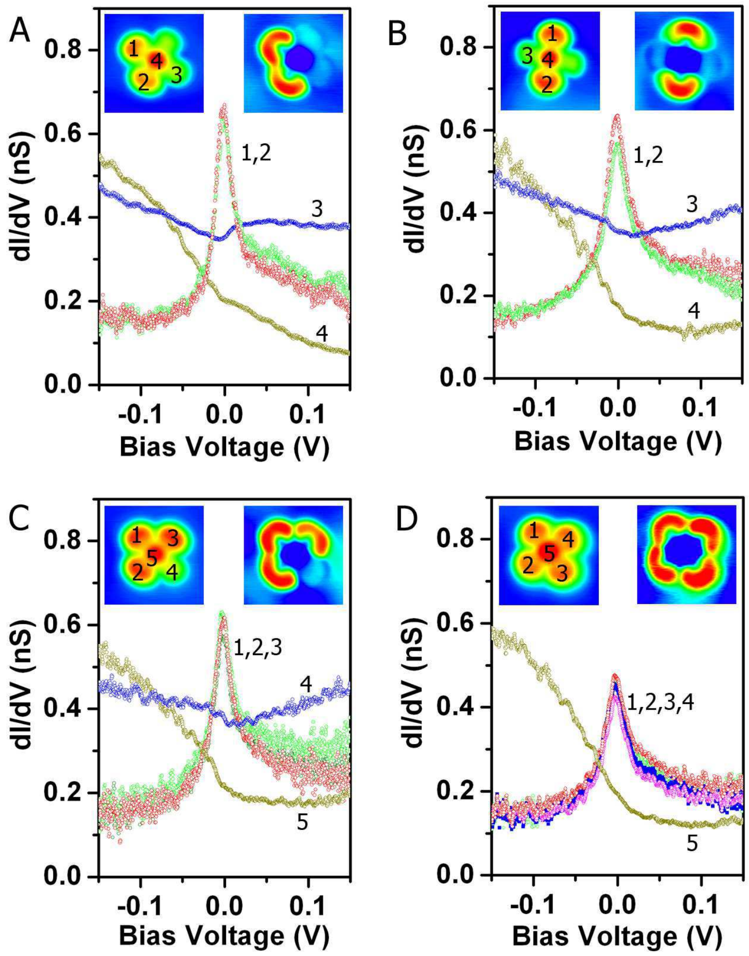

Supplementary Figure 2: The dI/dV spectra of (A) a K_2, (B) a $K_{2'}$, (C) a K_3 and (D) a K_4 measured by placing the STM tip at the marked points. The insets in the upper-left and upper-right corners of each panel are the topographic image and simultaneously acquired dI/dV map, respectively. Image size: $2.5{\times}2.5\,\mathrm{nm}^2$.

Supplementary Note 3: Manipulation of a K_1 complex on the Au(111) surface

By manipulating the STM tip, a K_1 can be moved to the proximity of a bare Co adatom or another K_1. Supplementary Figure 3(A) and (B) demonstrate the process that a K_1 is moved toward a target Co adatom. The distance between the captured Co adatom in K_1 and a bare Co adatom can reach 1.05 and 0.85 nm, respectively. Similarly, Supplementary Figure 3(C) and (D) demonstrate the process that a K_1 is moved toward another target K_1 complex. The distance between two captured Co adatom in K_1 complexes can reach 0.96 and 0.84 nm, respectively. The variation of T_K of the target Co adatom and the target K_1 complex during the manipulation processes are exhibited in Fig. 2(b) in the main text.

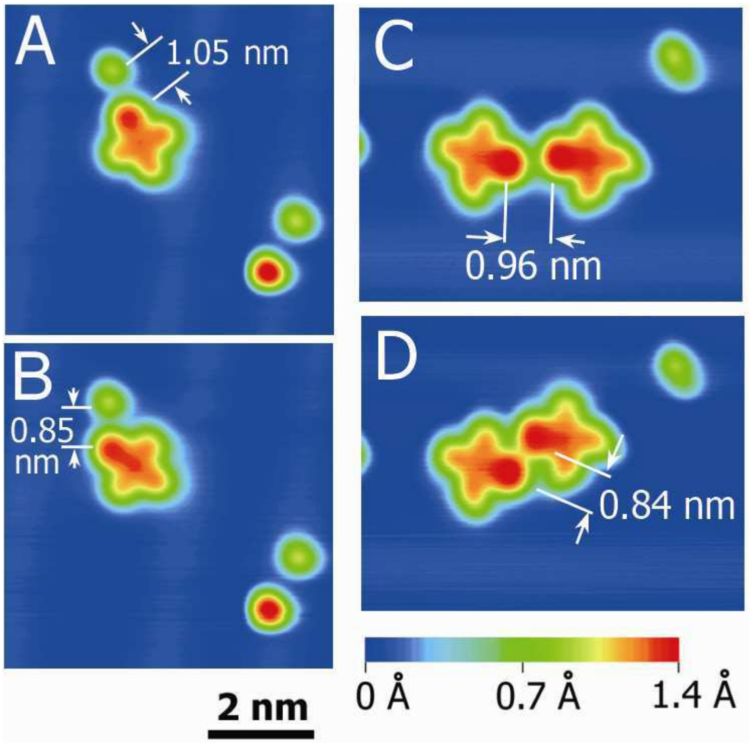

Supplementary Figure 3: (A) and (B) are topographic images of a K_1 complex approaching a bare Co adatom on the Au(111) surface. (C) and (D) are topographic images of a K_1 complex approaching another K_1 on the Au(111) surface.

Supplementary Note 4: Experimental determination of Kondo temperature and the influence of modulation voltage

In our experiments, the Kondo temperature of a K_n/Au(111) composite is measured by taking the following procedure. First, the dI/dV versus V spectrum is measured at a fixed temperature T. Then, the differential conductance spectrum is fit to the Fano line shape, from which the half width at half maximum (HWHM) of the Kondo conductance peak centered at zero bias (Γ) is extracted. Finally, Kondo temperature is determined by using the relation of Fermi liquid theory[1] with the thermal and instrument-induced broadening deconvoluted from the measured spectra:[2–5]

$$k_{\mathrm{B}}T_{\mathrm{K}} \approx \sqrt{\Gamma^2 - (\lambda k_{\mathrm{B}}T)^2 - (0.87\, eV_{\mathrm{m}})^2}. \tag{1}$$

Here, T is the environmental temperature; the coefficient λ is equal to π in the original Fermi liquid theory,[6] while it has assumed an empirical value of 2.7 in previous experiments;[4] and the last term in the square-root accounts for the instrument-induced peak broadening caused by the sinusoidal modulation voltage of the amplitude V_{m}.[7]

The influence of instrument on the measured Kondo temperature has been examined carefully in the literature.[2,3] Since our experiments are conducted at a low temperature $T = 5\,\mathrm{K}$ by using a small modulation voltage (peak-to-peak value is $2\,\mathrm{mV}$ and hence the amplitude is $V_{\mathrm{m}} = 1\,\mathrm{mV}$), the thermal fluctuations ($k_{\mathrm{B}}T$) and the modulation voltage (V_{m}) give rise to a rather minor contribution to the total broadening Γ. For instance, the measured broadening is $\Gamma = 10.6\,\mathrm{meV}$ for the K_1/Au(111) composite, we thus have $k_{\mathrm{B}}T_{\mathrm{K}} \approx \sqrt{\Gamma^2 - (1.16\,\mathrm{meV})^2 - (0.87\,\mathrm{meV})^2} = 10.5\,\mathrm{meV}$, i.e., the influence of thermal and instrument-induced broadening amounts to a small correction of T_{K} of only about $1.2\,\mathrm{K}$. Even so, we have explicitly accounted for these minor contributions in Fig. 2 of the main text.

Supplementary Note 5: Geometrical and electronic structures of the CoPc molecular mold

An isolated CoPc molecule has a planar geometry with the $D_{4\mathrm{h}}$ symmetry. It contains a Co ion at its center, which is coordinated with four nitrogen atoms and enclosed by an aromatic macrocycle. In the ground state, the molecule has a net spin of $S = \frac{1}{2}$ due to a spin-unpaired electron occupied the d_{z^2} orbital of the central Co ion. When the CoPc molecule is adsorbed on the Au(111) surface, the molecule structure does not undergo a significant change and remains almost planar.

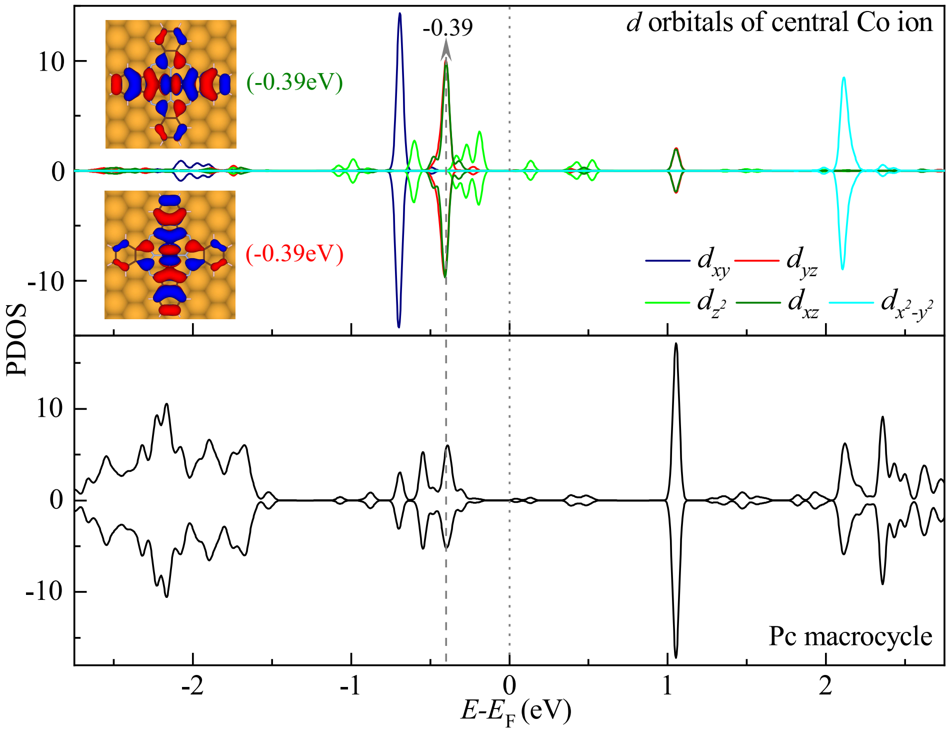

Supplementary Figure 4: PDOS of d orbitals on the central Co ion (upper panel) and that of the Pc macrocycle (lower panel) in a CoPc molecular mold on top of the Au(111) surface. The Fermi energy E_{F} is set to zero. The insets display two delocalized Kohn-Sham orbitals at the energy $E = -0.39\,\mathrm{eV}$.

Supplementary Figure 4 displays the calculated projected density of states (PDOS) of the d orbitals on the Co ion and that of the phthalocyanine (Pc) macrocycle in a CoPc mold placed on top of the Au(111) surface. The PDOS of majority-spin and minority-spin electrons are nearly identical, indicating the d electrons on the central Co ion are spin-unpolarized.

Two delocalized Kohn-Sham orbitals of the CoPc molecular mold are shown in the insets of Supplementary Figure 4. These orbitals are of crucial importance to the long-range superexchange (SE) interaction between the captured Co adatoms in the para positions of a $K_{2'}$ or a K_4 complex.

Supplementary Note 6: Geometrical and electronic structures of the K_n/Au(111) composites

Supplementary Figure 5 displays the optimized structure of a K_1 complex adsorbed at different sites on the Au(111) surface. The molecular plane of CoPc is parallel to the gold surface, with the six-member ring of each isoindole unit located right above a captured Co adatom. Such a geometry remains almost unchanged upon moving the complex to different sites or domains on the Au surface.

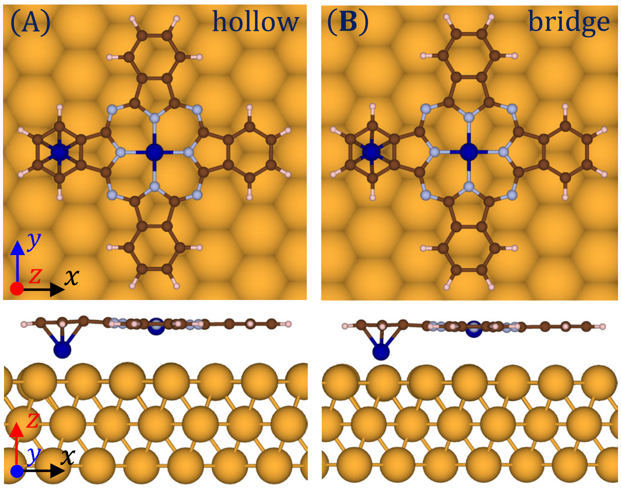

Supplementary Figure 5: Top and side views of the optimized structure of a K_1 complex absorbed at (A) a hollow site and (B) a bridge site on the Au(111) surface.

For all the K_n ($n = 1, 2, 2', 3, 4$) complexes, our density functional theory (DFT) calculations affirm that the spin-unpaired electrons reside predominantly on the captured Co adatoms, with only a tiny amount on the Co ion of the CoPc mold.

Supplementary Figure 6 shows the PDOS of different parts of a K_1 complex with the captured Co adatom adsorbed at a hollow site on the Au(111) surface. Clearly, the PDOS of the d orbitals on the captured Co adatom exhibits conspicuous spin polarization. Influenced by the captured Co adatom, the PDOS of the Co ion in the CoPc mold also exhibits weak spin polarization, and so does that of the Pc macrocycle.

Supplementary Table 1 lists the electron occupation numbers of the d orbitals on the captured Co adatom in a K_1/Au(111) composite, along with their contributions to the local spin moment S_z. In distinct contrast to the Co ion in the CoPc mold which has a negligible S_z, the captured Co adatom has an appreciable value of $S_z \approx 0.5$, which originates mainly from the d_π (d_{xz} and d_{yz}) orbitals. Thus, the Co adatom is considered to be in a local $S = \frac{1}{2}$ state.

Note that a halfly occupied orbital often refers to the scenario that the orbital is occupied by a spin-up electron with no spin-down electron occupancy, and so the overall spin moment is $S_z = \frac{1}{2}$. In contrast, for the captured Co adatom in a K_1, each of the d_π orbitals is occupied

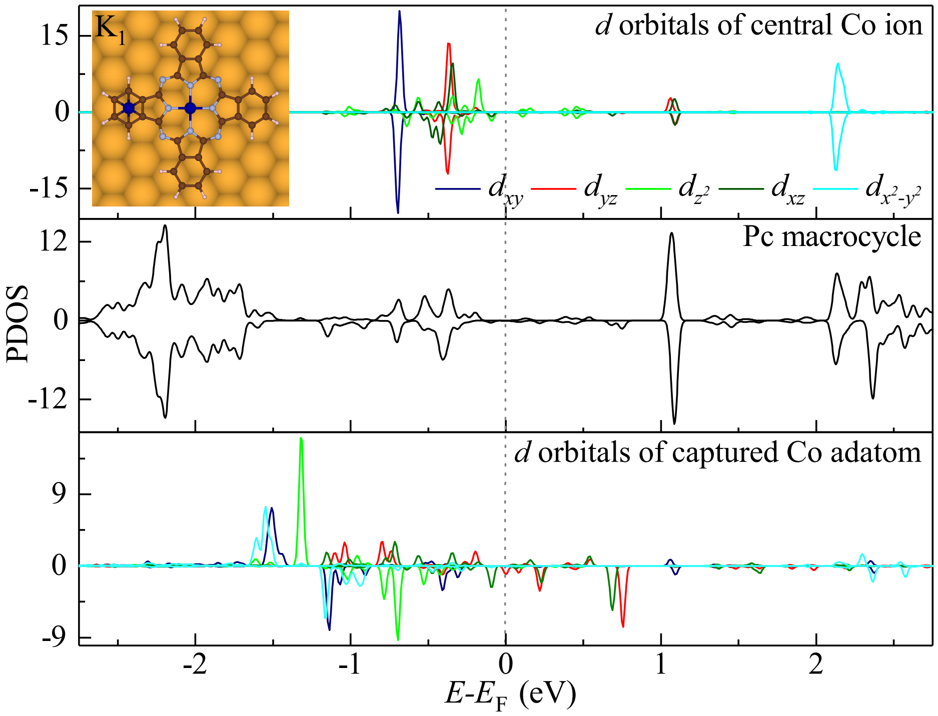

Supplementary Figure 6: Upper panel: PDOS of d orbitals on the Co ion of the CoPc mold in a K_1/Au(111) composite. Middle panel: PDOS of the Pc macrocycle. Lower panel: PDOS of d orbitals on the captured Co adatom. The Fermi energy E_F is set to zero. The inset in the upper panel displays the geometric structure of the K_1/Au(111) composite.

by roughly one spin-up electron but also by half a spin-down electron; see Supplementary Table 1 as well as the inset of Fig. 3(b) in the main text. Because of the fractional (nonzero) occupation of spin-down electrons, each d_π orbital contributes only about $s_z = 0.21$ to the local spin moment, and it is the sum of s_z of all the five Co d orbitals that amounts to the spin moment ($S_z = \frac{1}{2}$) of a purely halfly occupied orbital.

For the $K_{2'}$/Au(111) composite in which two Co adatoms locate at the para positions of the Pc ligand, the local spins on the two distantly separated Co adatoms can be parallel or anti-parallel to each other, corresponding to a ferromagnetic (FM) or antiferromagnetic (AFM) spin-state of the composite, respectively.

Because of the long-range SE interaction between the two local spins mediated by the delocalized Kohn-Sham orbitals on the CoPc mold (see Supplementary Figure 4), there is a finite energy gap between the FM and AFM spin-states, $J_{\text{eff}} = E_{\text{AFM}} - E_{\text{FM}} \approx \gamma \frac{\Delta_{\text{SE}}^2}{U+\Delta\epsilon} = 14\,\text{meV}$, where $\Delta\epsilon = |\epsilon_d - \epsilon_{\text{channel}}| = 0.205\,\text{eV}$, $\Delta_{\text{SE}} = 0.123\,\text{eV}$, $U = 0.88\,\text{eV}$ (see Supplementary Note 9 below), and $\gamma = 1$. It is worth mentioning that such a value of J_{eff} is but a crude estimate, because of the potential uncertainty in the prefactor γ.[8]

DFT calculation using the PBE functional yields an energy gap of $J_{\text{eff}} = 4\,\text{meV}$, and the spin density distributions corresponding to both the FM and AFM spin-states of the $K_{2'}$/Au(111) composite are shown in Supplementary Figure 7. Since the GGA functional

Supplementary Table 1: Electron occupation numbers for the d orbitals on the Co ion in the CoPc mold and on the captured Co adatom in a K_1/Au(111) composite. Here, $n_{\rm occ} = n_\uparrow + n_\downarrow$ and $S_z = \frac{1}{2}(n_\uparrow - n_\downarrow)$, with $n_\uparrow$ and $n_\downarrow$ being the majority-spin and minority-spin occupation numbers, respectively.

Co ion in CoPc	d_{xy}	d_{yz}	d_{z^2}	d_{xz}	$d_{x^2-y^2}$	Total
$n_\uparrow$	0.919	0.808	0.745	0.790	0.419	3.680
$n_\downarrow$	0.930	0.802	0.726	0.777	0.415	3.650
n_{occ}	1.849	1.610	1.471	1.566	0.834	7.330
S_z	-0.006	0.003	0.009	0.006	0.002	0.015
Co adatom	d_{xy}	d_{yz}	d_{z^2}	d_{xz}	$d_{x^2-y^2}$	Total
$n_\uparrow$	0.819	0.852	0.918	0.802	0.819	4.210
$n_\downarrow$	0.737	0.355	0.876	0.464	0.737	3.169
n_{occ}	1.556	1.207	1.794	1.266	1.556	7.379
S_z	0.041	0.249	0.021	0.169	0.041	0.521

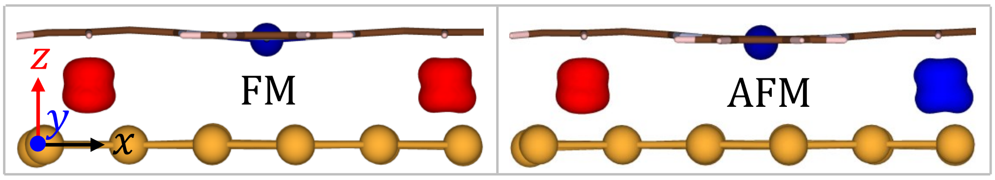

Supplementary Figure 7: Spin density distribution of a $K_{2'}$/Au(111) composite in the ferromagnetic (left panel) and antiferromagnetic (right panel) states. The value of isosurface is ± 0.05 Å^{-3}.

may suffer from static correlation error,[9] calculation of $J_{\rm eff}$ by more accurate *ab initio* quantum chemistry methods is appealing. This is however rather challenging because of the large size of the $K_{2'}$/Au(111) composite.

Supplementary Note 7: Influence of adsorption site on the local spin state of captured Co adatom

Supplementary Figure 8(A) depicts the PDOS of d_π (d_{xz} and d_{yz}) orbitals of a bare Co adatom adsorbed at a hollow site and a bridge site on the Au(111) surface, and there is an appreciable distinction in the line shape of PDOS between these two sites. In contrast, for a captured Co adatom in a K_1, the PDOS of d_π orbitals exhibit a minor change upon the variation of adsorption sites; see Supplementary Figure 8(B). This indicates that the strong bonding between the Co d_π orbitals and the conjugated π-orbitals of the isoindole unit has a predominant influence on the local electronic structure of the Co adatom. Consequently, regularized by the d_π-π bonding interaction, the local electronic structure of the Co adatom is hardly affected by the chemical environment beyond the CoPc molecular mold. It should be mentioned that the top-site adsorption is also explicitly considered in the calculation, but the captured Co adatom always relaxes to a hollow site through geometric optimization.

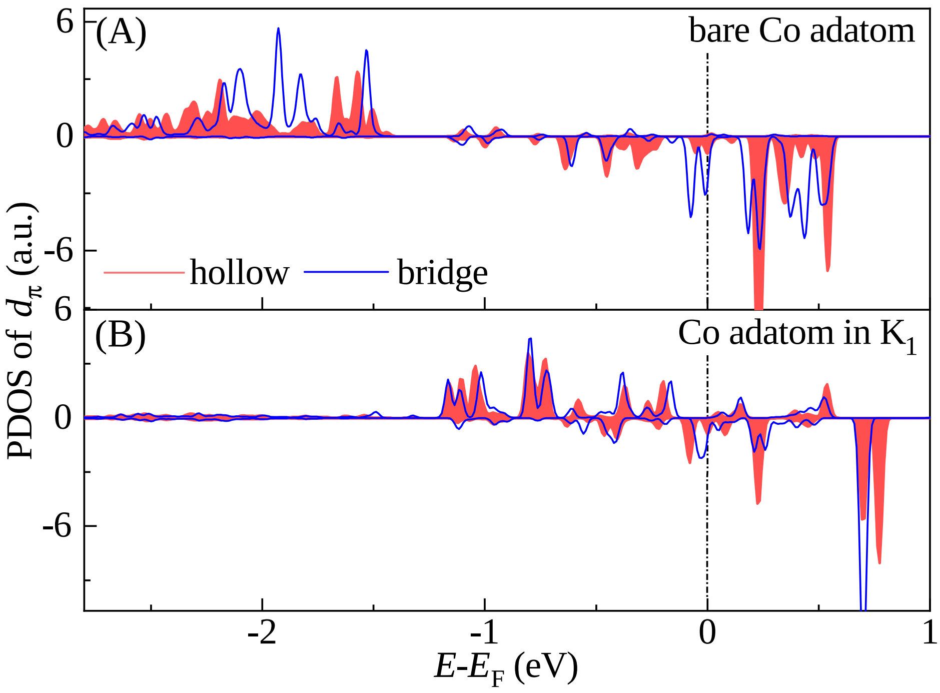

Supplementary Figure 8: PDOS of d_π (d_{xz} and d_{yz}) orbitals of (A) a bare Co adatom and (B) a captured Co adatom in a K_1 complex adsorbed at a hollow site and a bridge site on the Au(111) surface. The positive and negative values correspond to the majority-spin and minority-spin electrons, respectively. The vertical dash line indicates the position of the Fermi energy E_F.

Supplementary Note 8: Influence of surrounding environment on the local spin state of Co adatom

The influence of spin-orbit coupling (SOC) on the electronic structures of K_n/Au(111) composites is examined by turning on non-collinear spin in the DFT calculation. It is found that aligning the local spin in different magnetization directions results in a rather small variation in the total energy (less than 0.3 meV per Co adatom), indicating that the SOC has a negligible effect on the local electronic structures of the K_n complexes. This is also consistent with the designation of a local $S = \frac{1}{2}$ state for each captured Co adatom.

In a K_n/Au(111) composite, each Co adatom interacts with its surrounding environment, which consists of an isoindole ligand of the CoPc molecular mold and the gold substrate. In the following, we will elaborate on how the environment affects the local electronic structure of the Co adatom. In particular, we scrutinize the evolution of the PDOS of d_{xz} orbital, one of the d_π orbitals which contribute predominantly to the formation of Kondo states.

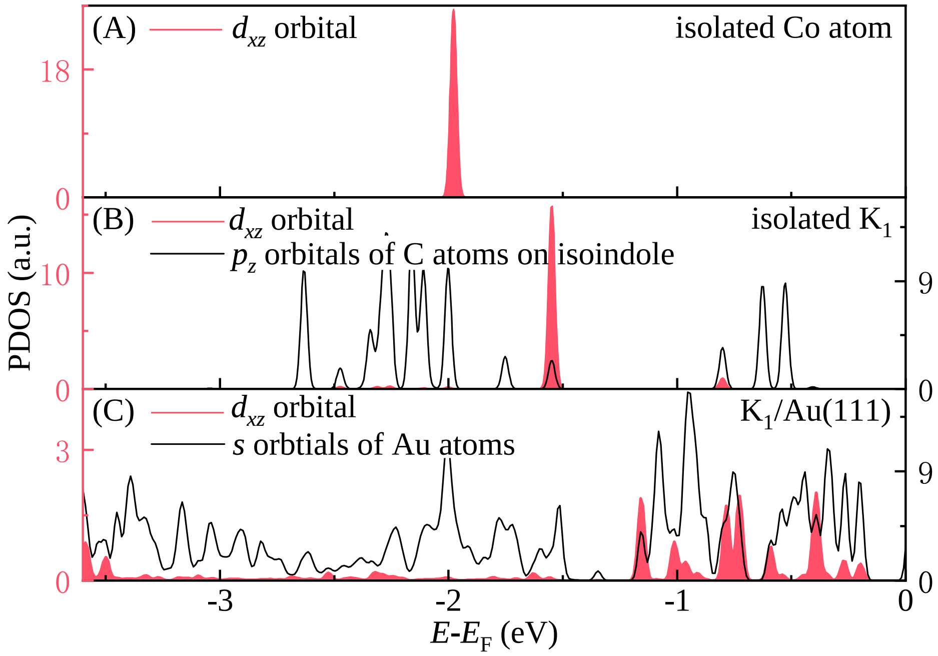

Supplementary Figure 9: (A) PDOS of the d_{xz} orbital of an isolated Co atom. (B) PDOS of the d_{xz} orbital of the captured Co atom in an isolated K_1 complex. The PDOS of the p_z orbitals of the carbon atoms on the adjacent isoindole ligand is also depicted. (C) PDOS of the d_{xz} orbital of the Co adatom in a K_1/Au(111) composite. The PDOS of the s orbitals of the three nearest gold atoms in the Au(111) substrate is also depicted.

For an isolated Co atom, the PDOS of its d_{xz} orbital exhibits a sharp peak; see Supplementary Figure 9(A). This affirms that the d_{xz} orbital is an eigen energy level of the Co atom. In an isolated K_1 complex, the Co adatom binds to an isoindole ligand of the CoPc mold through the d_π-π bonding interaction. The isoindole ligand field results in the

rearrangement of the Co d orbitals and regularization of the local spin distribution (analogous to that depicted in Fig. 3(a) of the main text). On the other hand, it is evident from Supplementary Figure 9(B) that the PDOS of the d_{xz} orbital retains a single-peak structure – the main peak amounts to 90% of the total area. This suggests that the d_{xz} orbital of the Co atom largely preserves its atomic feature even under the influence of the ligand field.

Supplementary Figure 9(C) plots the PDOS of the d_{xz} orbital of the Co adatom in a K_1/Au(111) composite, whose line shape is distinctly different from those displayed in Supplementary Figure 9(A) and (B). The PDOS of the d_{xz} orbital is no longer a single peak. Instead, it has a rather scattered distribution over a wide energy range. Moreover, there is an apparent correspondence between the peak positions of the PDOS of Co d_{xz} orbital and those of s orbitals of the nearest gold atoms. Such a correspondence highlights a strong hybridization between the Co d_{xz} orbital and the Au s orbitals. It is this hybridization that leads to the formation of Kondo states at the K_1/Au(111) interface, since the latter is known to originate from the screening of local spin moment (contributed predominantly by the Co d_π orbitals) by the spins of itinerant electrons in the metal substrate (contributed by the Au s orbitals).

In general, the broadening (or splitting) of an atomic d orbital reflects its interaction with the surrounding environment. As shown in Supplementary Figure 6, the d_{xy}, $d_{x^2-y^2}$, and d_{z^2} orbitals of the Co adatom have much smaller broadening under the influence of the environment. Considering also the fact that these orbitals only present a minor contribution to the local spin moment (see Supplementary Table 1), we conclude that it is the d_π (d_{xz} and d_{yz}) orbitals that contribute predominantly to the formation of Kondo states.

As shown in Supplementary Figure 9, the splitting of the d_{xz} orbital is caused solely by the hybridization with the s orbitals of surrounding gold atoms. Therefore, the energy span of the split d_π orbitals characterizes the s-d hybridization strength (Δ_s),[10] which is a decisive factor of the strength of Kondo screening.[11] Conventionally, the magnitude of Δ_s is extracted as the HWHM for the broadened or split d orbital.[10,12] However, for the K_n/Au(111) composites in our study, the PDOS of the split d_π orbitals has a complicated line shape, and thus we propose to extract Δ_s as half of the full width of the split peaks; see Fig. 3(b) of the main text.

Supplementary Note 9: Parameters of Anderson impurity models for the K_n/Au(111) composites

The values of the Anderson impurity model (AIM) parameters, including ϵ_d, U, Δ_α, Ω_α and W_α, are extracted from the results of DFT calculation.[10,12,13]

- The energy of the impurity orbital is estimated as $\epsilon_d = \int \rho_d(\omega)\omega d\omega / \int \rho_d(\omega) d\omega$, where $\rho_d(\omega)$ is the PDOS of the impurity orbital. Here, each impurity orbital represents the sum of d_π (d_{xz} and d_{yz}) orbitals on a Co adatom. The electron occupation number on the impurity orbital is calculated by $n_d = \int_{-\infty}^{E_\mathrm{F}} \rho_d(\omega) d\omega$.
- W_α is estimated as the width of the energy bands of the STM tip ($\alpha = \mathrm{t}$) or the gold substrate ($\alpha = \mathrm{s}$) which have nonzero couplings to the d_π orbitals.
- Ω_α is taken to be the same as the chemical potential of the α-reservoir.
- The on-site electron-electron Coulomb interaction energy U is extracted as the energy difference between the centers of the spin-up and spin-down d_π orbitals.
- Δ_s is the hybridization strength between the impurity and substrate orbitals, as it represents the amplitude of the hybridization function $\Lambda_\mathrm{s}(\omega) \equiv \pi \sum_k |t_{kd}|^2 \delta(\omega - \epsilon_{k\mathrm{s}}) = \frac{\Delta_\mathrm{s}}{2} \frac{W_\mathrm{s}^2}{(\omega - \Omega_\mathrm{s})^2 + W_\mathrm{s}^2}$. In our experiments, the STM tip is in the tunneling regime, and we thus assume Δ_t has a small constant value ($\Delta_\mathrm{t} \ll \Delta_\mathrm{s}$).

  In the wide-band limit ($\Delta_\alpha \ll W_\alpha$), the spectral function of the impurity orbital is

$$\rho_d(\omega) = -\frac{1}{\pi}\mathrm{Im}[G_d^r(\omega)] \simeq \frac{1}{\pi}\frac{\Delta}{(\omega - \epsilon_d)^2 + \Delta^2}, \quad (2)$$

  where $G_d^r(\omega)$ is the retarded Green's function of the impurity orbital, and $\Delta = \Delta_\mathrm{s} + \Delta_\mathrm{t} \approx \Delta_\mathrm{s}$.

  Note that $\rho_d(\omega)$ is just the PDOS of the d_π orbitals of the captured Co adatoms, so Δ_s could be extracted as the HWHM of the broadened (or split) peak of $\rho_d(\omega)$, provided that $\rho_d(\omega)$ retains a single-peak structure. However, as shown in Supplementary Figure 9(C), $\rho_d(\omega)$ has a rather complicated line shape because of the strong s-d hybridization. Thus, Δ_s is extracted as the half width of the split peaks which constitute the major contribution to the local spin moment; see Fig. 3(b) in the main text. Numerically, the value of Δ_s is determined by cutting the split peaks with a sufficiently small threshold. For instance, the thresholds of 0.05 and 0.1 eV^{-1} result in $\Delta_\mathrm{s} = 0.261$ and 0.258 eV for the K_1/Au(111) composite, respectively. It is estimated that the uncertainty of the cutting threshold could cause an uncertainty of ~ 5 K in the predicted Kondo temperature.

- The SE interaction between the two spatially separated Co adatoms in the $K_{2'}$/Au(111) complex, Δ_{SE}, is taken as a tunable parameter. Its value is finally settled by comparing the Kondo temperature T_K determined by the HEOM calculation carried out on a two-orbital AIM to the experimental measurement; see Fig. 4 in the main text and Supplementary Note 10 below.

The values of all the AIM parameters for the K_n/Au(111) composites are listed in Supplementary Table 2.

Supplementary Table 2: Values of the AIM parameters for the K_n/Au(111) composites (in units of eV).

K_n	ϵ_d	U	Δ_s	Δ_{SE}	Δ_t	W_α ($\alpha = s, t$)
K_1	-0.585	0.906	0.258	0	0.01	5.5
K_2	-0.601	0.909	0.268	0	0.01	5.5
$K_{2'}^*$	-0.575	0.880	0.273	0	0.01	5.5
$K_{2'}$	-0.575	0.880	0.273	0.123	0.01	5.5
K_3	-0.588	0.896	0.278	0	0.01	5.5
K_4	-0.631	0.924	0.288	0	0.01	5.5

Supplementary Note 10: Theoretical determination of Kondo temperatures of K_n/Au(111) composites

We calculate the differential conductance spectra (dI/dV versus V) of the K_n/Au(111) composites by applying the hierarchical equations of motion (HEOM) method and adopting a single-orbital AIM. The results are compared with the experimental measurements in Supplementary Figure 10(A). The agreement between the theoretically obtained line shape and the experimental counterpart is remarkable.

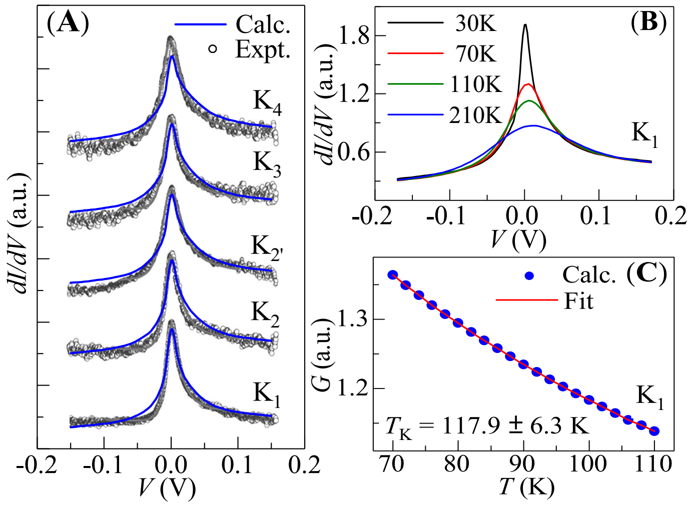

Supplementary Figure 10: (A) Calculated dI/dV versus V for the K_n/Au(111) composites by adopting the single-orbital AIM. Limited by the computer resources at our disposal, here the temperature adopted in the calculation is 30 K, which is somewhat higher than the experimental value of 5 K. (B) Calculated dI/dV spectra of a K_1/Au(111) at different temperatures. (C) Calculated zero-bias conductance as a function of temperature and the best fit to the empirical scaling relation of Supplementary Equation (3).

It has been demonstrated both theoretically[14] and experimentally[15,16] that the zero-bias conductance $G \equiv (\frac{dI}{dV})_{V=0}$ of a Kondo impurity obeys the following empirical scaling relation:

$$G(T) = G_0\left[1 + \left(\frac{T}{T_{\rm K}}\right)^2 \left(2^{\frac{1}{s}} - 1\right)\right]^{-s} + g_{\rm b}. \tag{3}$$

Here, T is the environmental temperature, G_0 is the conductance at $T \to 0$, $g_{\rm b}$ is the background conductance due to the electron transport through the non-Kondo states, and s is a parameter whose value depends on the local spin state of the impurity. Particularly, $s = 0.22$ is adopted as suggested by previous calculations on a spin-$\frac{1}{2}$ impurity.[14–16] The Kondo temperature $T_{\rm K}$ can thus be determined by fitting the calculated G versus T to Supplementary Equation (3). As an example, Supplementary Figure 10(B) shows the calculated dI/dV spectra of the K_1/Au(111) composite at different T, and the best fit of $G(T)$ is displayed in Supplementary Figure 10(C).

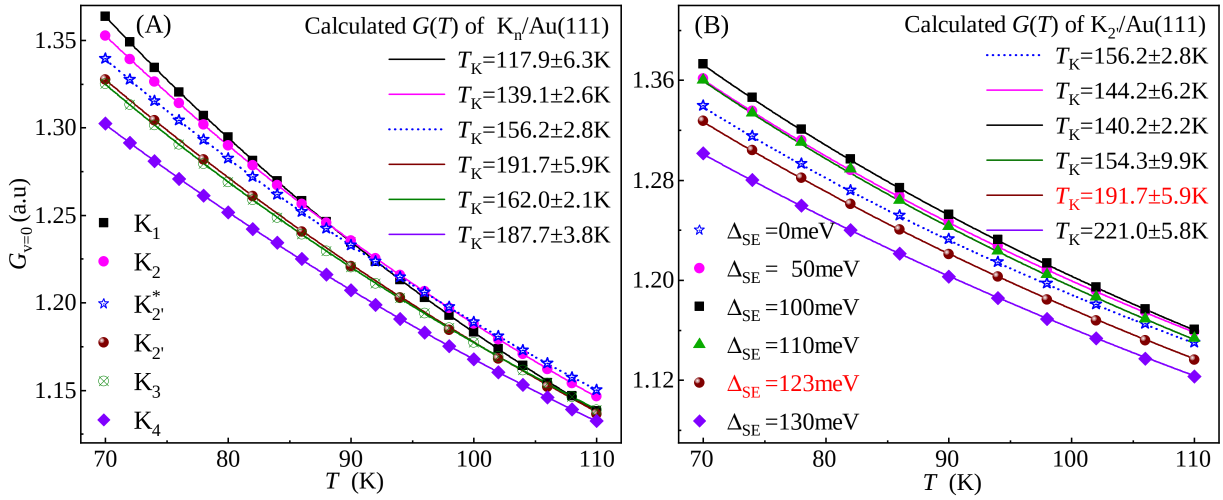

Supplementary Figure 11: (A) Calculated zero-bias conductance $G(T)$ of all the K_n/Au(111) composites by adopting a single-orbital AIM, except that for the $K_{2'}$/Au(111) composite a two-orbital AIM is adopted. The lines are the best fit of $G(T)$ to the empirical scaling relation of Supplementary Equation (3). The simulated T_K of the composites and their standard deviations are given in the upper-right corner. $K_{2'}^*$ denotes a reference system represented by a single-orbital AIM, but with all the parameters assuming the same values as those of $K_{2'}$. (B) Calculated zero-bias conductance $G(T)$ of the $K_{2'}$/Au(111) composite by adopting a two-orbital AIM with different values of Δ_{SE}. The lines are the best fit of $G(T)$ to Supplementary Equation (3). The simulated T_K of the composites and their standard deviations are given in the upper-right corner.

Supplementary Figure 11(A) shows the calculated zero-bias conductance of all the K_n/Au(111) composites at various temperatures and their best fit to Supplementary Equation (3). The resulting T_K are given in Fig. 2(d) of the main text, and also in the top-right corner of Supplementary Figure 11(A). It is remarkable that the theoretically determined T_K by a single-orbital AIM almost coincide with the experimental values for K_n $(n = 1, 2, 3, 4)$ except for $K_{2'}$. If the $K_{2'}$/Au(111) composite is represented by a single-orbital AIM (such a reference system is denoted as $K_{2'}^*$), its T_K is predicted to be 157 K, which is only slightly higher than the T_K of a K_2 but considerably lower than the experimental value of 191 K. This indicates a single-orbital AIM is inadequate to address the Kondo effect in the $K_{2'}$/Au(111) composite, and the long-range interaction between the two remote Co adatoms has a non-trivial influence on the Kondo states at the $K_{2'}$/Au(111) interface.

We then calculate $G(T)$ for the $K_{2'}$/Au(111) composite by adopting a two-orbital AIM with different values of Δ_{SE}; see Supplementary Figure 11(B). The resulting T_K are given in Fig. 4 of the main text and in the top-right corner of Supplementary Figure 11(B). In particular, the theoretical data of $\Delta_{SE} = 123\,\text{meV}$ recover the experimental value of $T_K = 191\,\text{K}$; see also Fig. 2(d) of the main text.

In addition to the empirical scaling relation of the zero-bias conductance versus temperature, Kondo temperature has also been determined by using the Fermi liquid theory, i.e., the relation of Supplementary Equation (1). In fact, this is how the experimental value of T_K are

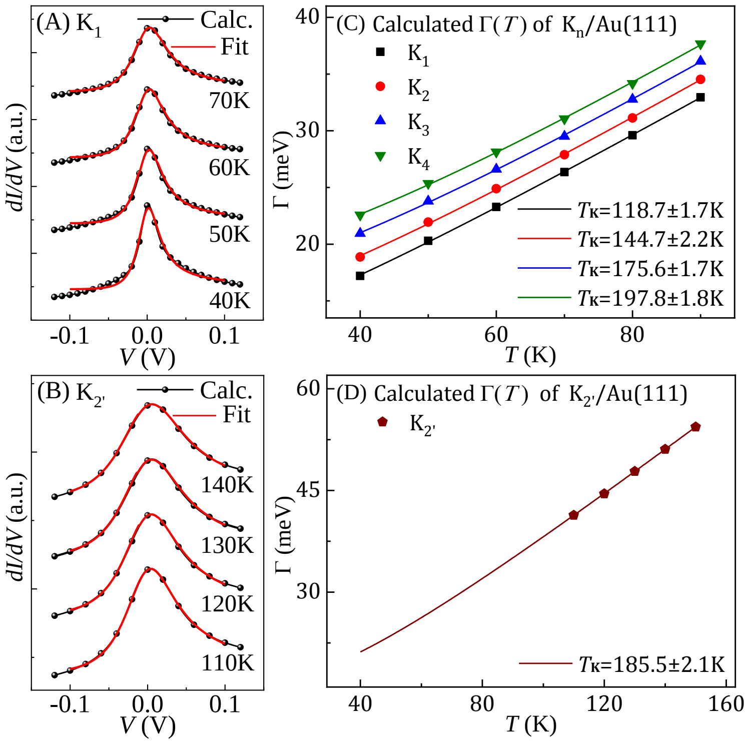

Supplementary Figure 12: Calculated dI/dV spectra for the (A) K_1/Au(111) and (B) $K_{2'}$/Au(111) composites at different temperatures. The scattered data in (A) and (B) are obtained by employing the HEOM method and by adopting the single- and two-orbital AIMs, respectively. The lines are best fit of the calculated data to a Fano function; see Eq. (1) of the main text. The widths of the dI/dV spectral peaks, $\Gamma(T)$, are displayed in (C) for $n = 1, 2, 3, 4$, and in (D) for $n = 2'$, respectively. The corresponding T_K are extracted by fitting $\Gamma(T)$ to Supplementary Equation (1), and their values are given in the bottom-right corner of (C) and (D).

determined in this work. Theoretical analysis based on Supplementary Equation (1) is also carried out, which is computationally rather expensive, because it requires the calculation of dI/dV for the whole low bias region, rather than just the zero-bias conductance.

Supplementary Figure 12(A) and (B) depict the calculated dI/dV spectra for the K_1/Au(111) and $K_{2'}$/Au(111) composites at different temperatures, respectively. Apparently, the conductance peak gets broadened as the environmental temperature increases, and the calculated data fit well to a single Fano function; see Eq. (1) of the main text. The Kondo temperature T_K is extracted by fitting the width of the peak, $\Gamma(T)$, to the relation of Supplementary Equation (1) while taking α to be a tunable parameter; see the bottom-right corner of Supplementary Figure 12(C) and (D). Such determined T_K agree consistently with those obtained via the empirical scaling relation; see the direct comparison displayed in Fig. 2(d) of the main text.

The remarkable agreement between the two sets of theoretical data, as well as between the theoretical and experimental results, justifies the AIMs adopted to represent the K_n/Au(111) composites, and also affirms the accuracy of the DFT+HEOM calculations.

Supplementary Note 11: Binding energy of Co adatoms in the K_n/Au(111) composites

The average binding energy between a captured Co adatom and its surrounding environment in a K_n/Au(111) composite is defined by $E_b \equiv (E_{K_n/Au(111)} - E_{CoPc/Au(111)} - nE_{atom})/n$. Here, n is the number of captured Co adatoms, E_{atom} is the energy of an isolated Co atom, and $E_{K_n/Au(111)}$ and $E_{CoPc/Au(111)}$ are the total energies of the K_n/Au(111) and CoPc/Au(111) composites, respectively.

Supplementary Table 3 presents the evolution of E_b with the increasing n. The average binding energy for the captured Co adatoms in a K_n/Au(111) composite relative to that in a K_1/Au(111) composite is defined as $\Delta E_b(K_n) \equiv E_b(K_n) - E_b(K_1)$. Obviously, the magnitude of ΔE_b increases with the enlarging n, indicating a stronger coupling with the environment; see also Supplementary Figure 13. It is consistent with the result of Δ_s versus n for the K_n/Au(111) composites. For a $K_{2'}$ complex, the average binding energy E_b for the captured Co adatoms is somewhat larger than its counterpart in a K_2.

Supplementary Table 3: Average binding energy for the captured Co adatoms and the average distances between a captured Co adatom and its neighboring Au (R_{Co-3Au}) and C (R_{Co-6C}) atoms in the K_n/Au(111) composites. All energies are in units of eV, and all distances are in units of Å. Note that spin-orbit coupling is explicitly included.

K_n	$E_{K_n/Au(111)}$	$E_{CoPc/Au(111)}$	E_{atom}	E_b	ΔE_b	R_{Co-3Au}	R_{Co-6C}
K_1	-1061.6344	-1055.1771	-0.7710	-5.6863	0	2.5443	2.1091
K_2	-1068.1163	-1055.1771	-0.7710	-5.6986	-0.0123	2.5482	2.1068
$K_{2'}$	-1068.1357	-1055.1771	-0.7710	-5.7083	-0.0220	2.5649	2.1052
K_3	-1074.6402	-1055.1771	-0.7710	-5.7167	-0.0304	2.5493	2.1057
K_4	-1081.1479	-1055.1771	-0.7710	-5.7217	-0.0354	2.5497	2.1049

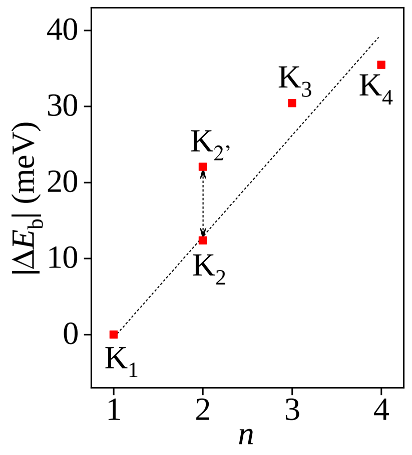

Supplementary Figure 13: The magnitude of E_b versus n for the captured Co adatoms in K_n/Au(111) composites. The dashed line is a guide for the eyes.

Supplementary Note 12: Experimental construction and measurement of K_n(CuPc) and K_n(H_2Pc) complexes

We have replaced the CoPc by CuPc and H_2Pc molecules to construct similar K_n(CuPc) and K_n(H_2Pc) complexes on the Au(111) surface. Supplementary Figure 14 shows the topographic images of those complexes and their dI/dV spectra measured by placing the STM tip on top of the captured Co adatoms. The lines shapes of the dI/dV spectra much resemble those of K_n(CoPc), and the Kondo peak at zero bias gets broader as n increase from 1 to 2 (or 2′); see Supplementary Figure 14(E) and (J). These results are generally similar to the ones from CoPc except for $K_{2'}$(H_2Pc) and $K_{2'}$(CuPc). The measured T_K of $K_{2'}$(CuPc) and $K_{2'}$(H_2Pc) are only slightly higher than their isomers K_2(CuPc) and K_2(H_2Pc) by 4∼5 K.

On the other hand, the construction of the K_n(CuPc) and K_n(H_2Pc) complexes with $n \geq 3$ was unsuccessful.

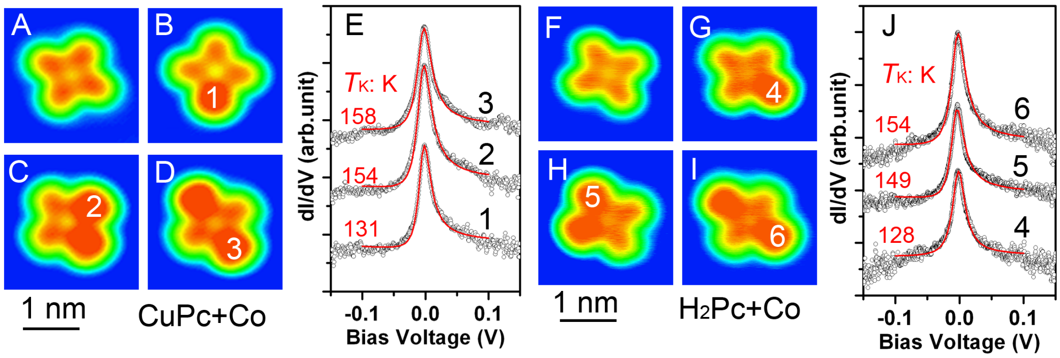

Supplementary Figure 14: (A)-(D) Topographic images of a pristine CuPc, a K_1(CuPc), a K_2(CuPc), and a $K_{2'}$(CuPc), respectively. (E) Kondo peaks measured by placing the STM tip on top of the captured Co adatoms. (F)-(I) Topographic images of pristine H_2Pc, K_1(H_2Pc), K_2(H_2Pc), and $K_{2'}$(H_2Pc), respectively. (J) Kondo peaks measured by placing the STM tip on top of the captured Co adatoms.

Supplementary Note 13: Electronic structures of $K_{2'}$(CuPc) and $K_{2'}$(H_2Pc) complexes

Supplementary Figure 15 depicts the PDOS of *d* orbitals that are supposedly relevant to the superexchange interaction between the two captured Co adatoms in a $K_{2'}$(CuPc) complex. The dashed line at -2 eV clearly indicates that the PDOS of *d* orbitals on the captured Co adatoms are energetically separated from that of the *d* orbitals on the central Cu ion. This confirms that the central Cu ion cannot bridge the long-range superexchange interaction between two captured Co adatoms in a $K_{2'}$(CuPc) complex. Similarly, the coupling channel for superexchange interaction also breaks down due to the lack of the central hub in a $K_{2'}$(H_2Pc) complex.

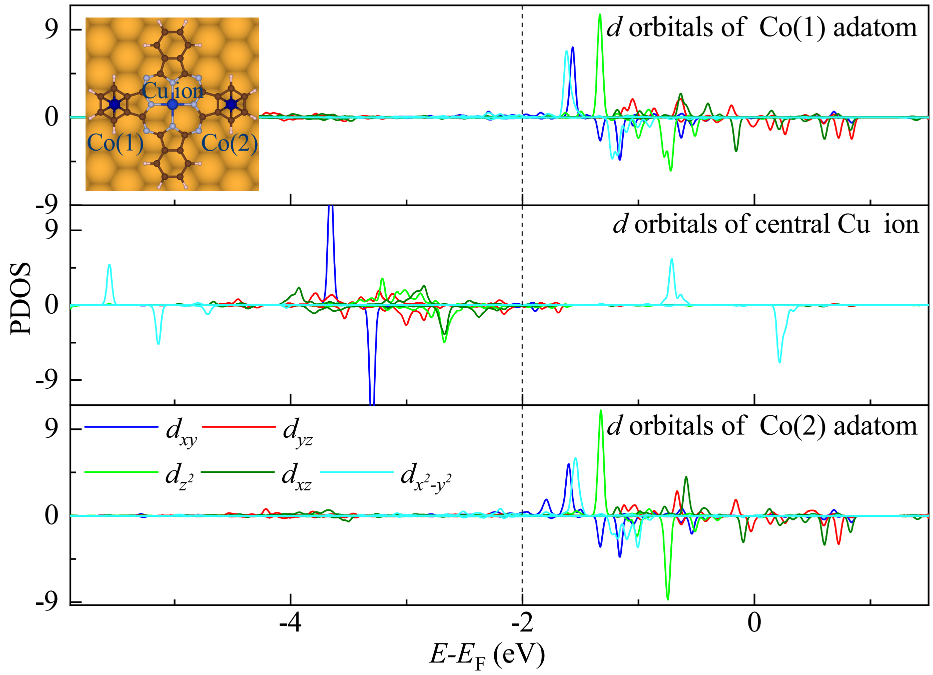

Supplementary Figure 15: Upper panel: PDOS of *d* orbitals on the captured Co(1) adatom in a $K_{2'}$(CuPc) complex. Middle panel: PDOS of *d* orbitals on the central Cu ion. Bottom panel: PDOS of *d* orbitals on the captured Co(2) adatom. The dashed line at -2 eV clearly indicates that the PDOS of *d* orbitals on the captured Co adatoms are energetically separated from that of the *d* orbitals on the central Cu ion. The inset in the upper panel is a structure model of the $K_{2'}$(CuPc)/Au(111) composite.

Supplementary Note 14: Existence/Absence of superexchange channel in the K_n/Au(111) composites

Supplementary Figure 16 displays the Kohn-Sham orbitals relevant to the SE interaction between the spatially separated Co adatoms in K_2/Au(111) and K_3/Au(111). As shown in the insets of Supplementary Figure 16(A) and (B), the delocalized orbitals on the CoPc mold appear to be much less connected than their counterparts in K_1/Au(111); see Supplementary Figure 4. Consequently, the long-range SE interaction is supposed to be much weaker in K_2/Au(111) and K_3/Au(111) composites.

Supplementary Figure 17 displays the delocalized Kohn-Sham orbitals in the $K_{2'}$/Au(111) and K_4/Au(111) composites. Because of the high structural symmetry of the atom-mold complex, the connectivity of the delocalized orbitals is well preserved in a $K_{2'}$ or a K_4. These orbitals play the role as the bridging channels for the long-range SE interaction. The channel in a K_4 complex is less effective than in a $K_{2'}$, because in the former the d_π orbitals on different Co adatoms are only partially connected by the channel; see remarks in Supplementary Note 15.

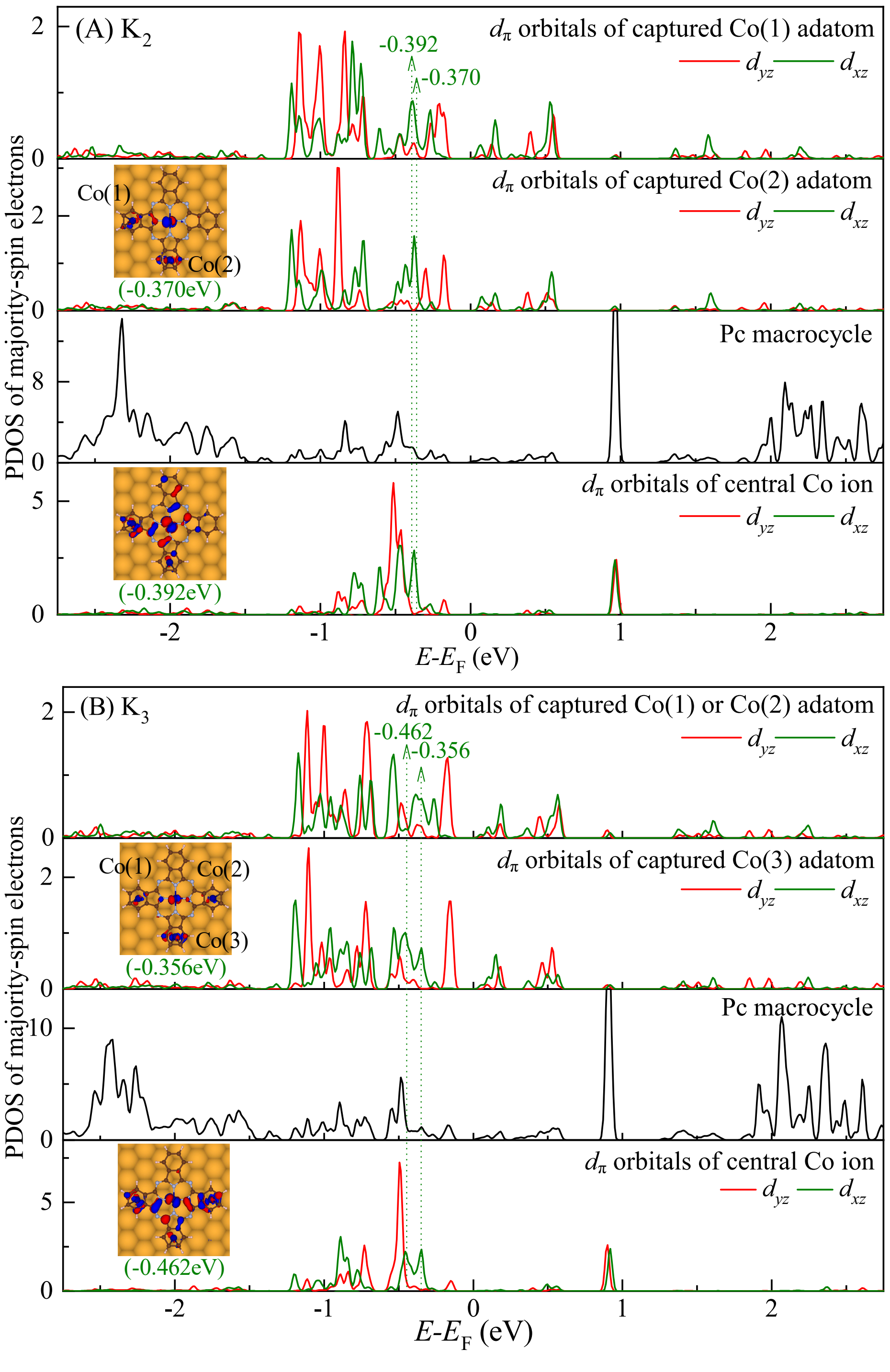

Supplementary Figure 16: PDOS of the d_π orbitals of the captured Co adatoms, the Pc macrocycle and the d_π orbitals of the central Co ion, for majority-spin electrons in (A) a K_2/Au(111) and (B) a K_3/Au(111) composite, respectively. The Fermi energy E_F is set to zero. The green dashed lines indicate the energies of the delocalized Kohn-Sham orbitals displayed in the insets. The isosurfaces of $\pm 0.002 Å^{-3}$ shaded in red and blue visualize the spatial distribution of the Kohn-Sham orbitals.

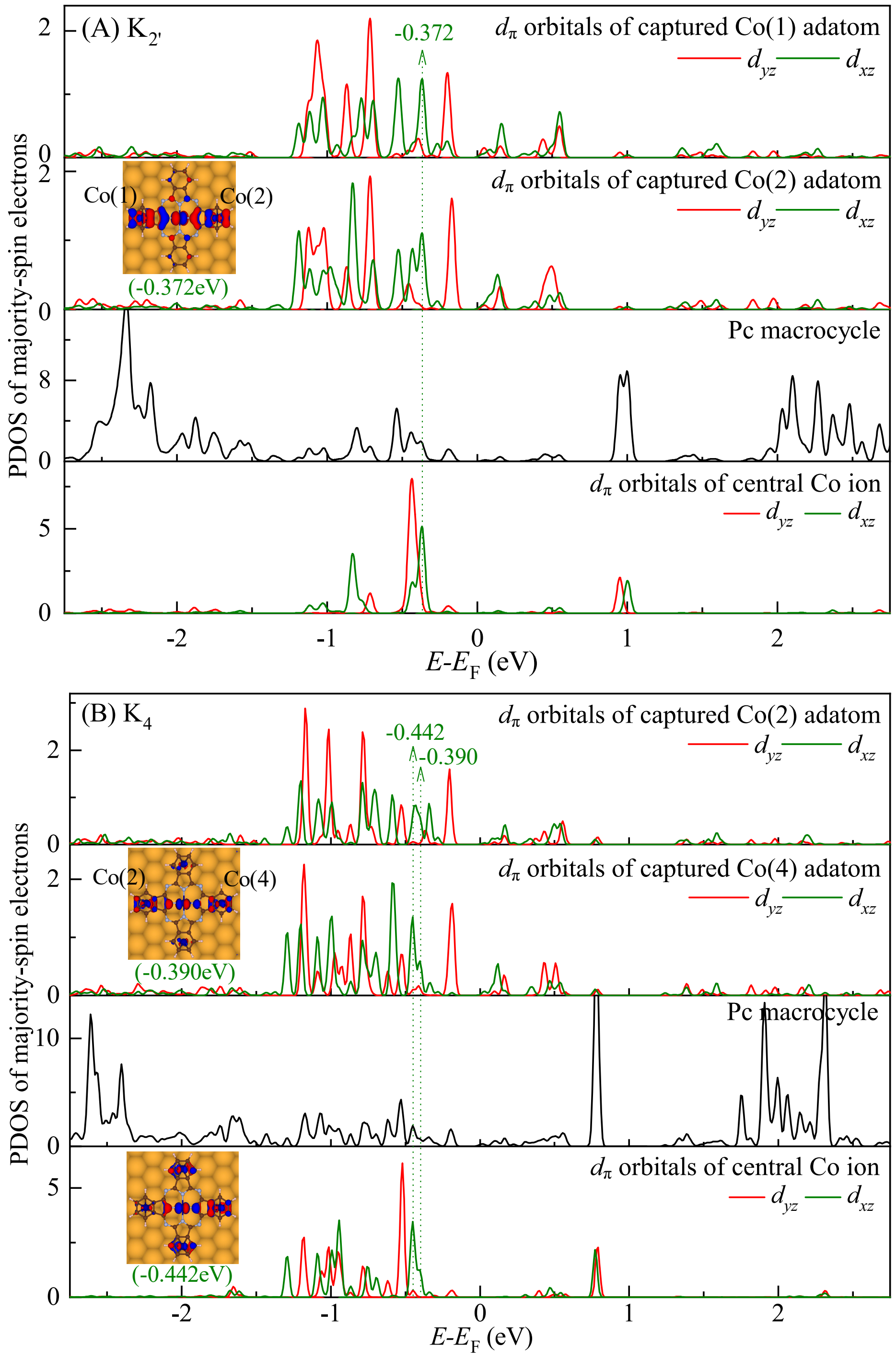

Supplementary Figure 17: PDOS of the d_π orbitals of the captured Co adatoms, the Pc macrocycle and the d_π orbitals of the central Co ion, for majority-spin electrons in (A) a $K_{2'}$/Au(111) and (B) a K_4/Au(111) composite, respectively. The Fermi energy E_F is set to zero. The green dashed lines indicate the energies of the delocalized Kohn-Sham orbitals displayed in the insets. The isosurfaces of $\pm 0.002 Å^{-3}$ shaded in red and blue visualize the spatial distribution of the Kohn-Sham orbitals.

Supplementary Note 15: Analysis on the superexchange interaction in $K_{2'}$/Au(111) and K_4/Au(111) composites

As described in Supplementary Note 6, each of the captured Co adatoms in a K_n/Au(111) composite is in a local $S = \frac{1}{2}$ state. Since it is the d_π orbitals that contribute predominantly to the Kondo screening, the local spin associated with the Kondo states is $S_z \simeq 0.42$.

For describing the Kondo states in the $K_{2'}$/Au(111) composite, we adopt a two-orbital AIM which involves an off-diagonal hybridization function, $\Lambda_{12}(\omega) = \frac{\Delta_{\rm SE}}{2}\frac{W_{\rm s}^2}{(\omega-\Omega_{\rm s})^2+W_{\rm s}^2}$, to characterize the long-range SE interaction between the two Co adatoms mediated by the CoPc mold. To verify that the SE interaction indeed results in an effective FM coupling between the two Co adatoms, we compute $S_{12} = \langle \hat{\mathbf{S}}_1 \cdot \hat{\mathbf{S}}_2 \rangle$ and $\langle \hat{\mathbf{S}}^2 \rangle$ as functions of $\Delta_{\rm SE}$ by using the HEOM method. Here, $\hat{\mathbf{S}}_i = \frac{1}{2}\sum_{ss'} \hat{c}_{is}^\dagger \boldsymbol{\sigma}_{ss'} \hat{c}_{is'}$ is the spin operator for the d_π orbitals of the ith Co adatom ($i = 1, 2$), with $\boldsymbol{\sigma}$ representing the set of Pauli matrices; and $\hat{\mathbf{S}} = \hat{\mathbf{S}}_1 + \hat{\mathbf{S}}_2$. The calculated results are shown in Supplementary Figure 18.

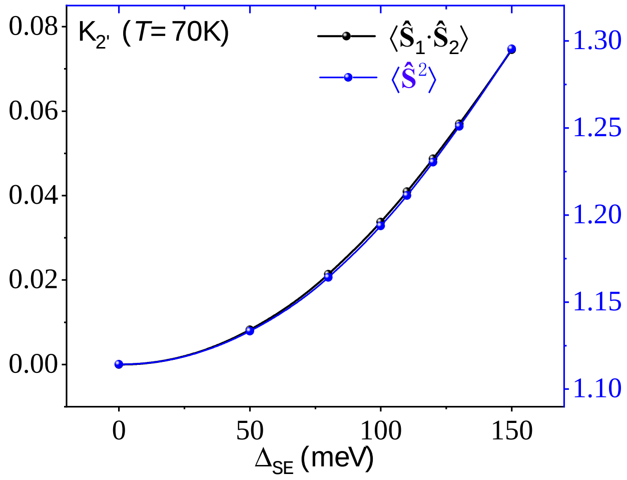

Supplementary Figure 18: Calculated S_{12} and $\langle \hat{\mathbf{S}}^2 \rangle$ versus $\Delta_{\rm SE}$ for a $K_{2'}$/Au(111) composite. A two-orbital AIM which explicitly involves $\Delta_{\rm SE}$ is adopted.

As shown in Supplementary Figure 18, the spin-spin correlation S_{12} increases monotonically from zero to a positive value with the increasing $\Delta_{\rm SE}$. This indicates that the two remotely separated local spins are independent of each other at $\Delta_{\rm SE} = 0$, while they are aligned toward a mutually parallel direction at a nonzero $\Delta_{\rm SE}$. It is noted that two lines almost overlap each other in Supplementary Figure 18. This is because the local spin moments on the Co adatoms (S_1 and S_2) remain almost constant upon the variation of $\Delta_{\rm SE}$. By using the relation $\langle \hat{\mathbf{S}}^2 \rangle = S(S+1)$, we have $\langle \hat{\mathbf{S}}_2^2 \rangle \approx \langle \hat{\mathbf{S}}_1^2 \rangle = S_1(S_1+1) = \sqrt{\big(\langle \hat{\mathbf{S}}^2 \rangle - 2S_{12}\big)/2}$, and thus $S_1 \approx S_2 = 0.4$. Therefore, the results yielded by the HEOM calculation on the two-orbital AIM agree closely with those of the DFT calculation, cf. Supplementary Table 1.

As shown in Fig. 4 of the main text, the Kondo temperature of a $K_{2'}$/Au(111) composite exhibits a non-monotonic dependence on the strength of SE interaction, i.e., $T_{\rm K}$ first de-

creases slowly with the increasing $\Delta_{\rm SE}$ to a minimal value, and then rises quickly as $\Delta_{\rm SE}$ increases further. Such a phenomenon can be understood by referring to a serially coupled two-orbital AIM that has been investigated previously.[17]

In a serially coupled two-orbital AIM, one impurity orbital is coupled to only one electron reservoir. The whole impurity is described by $H_{\rm imp} = \sum_{i=1,2}[\epsilon_i(\hat{n}_{i\uparrow} + \hat{n}_{i\downarrow}) + U\hat{n}_{i\uparrow}\hat{n}_{i\downarrow}] + t\sum_{\sigma=\uparrow,\downarrow}(\hat{d}^\dagger_{1\sigma}\hat{d}_{2\sigma} + \text{H.c.})$. Here, the last term represents the inter-orbital hopping (with t being the strength), which gives rise to an effective AFM coupling between the two orbitals, with the coupling strength being $J_t = \frac{4t^2}{U}$.[18] Numerical calculations have revealed that the Kondo effect exhibits a non-monotonic trend with the increase of t. Specifically, the Kondo effect is first enhanced as t increases from zero to a certain value, and then it gets suppressed as t increases further.[17] The mechanism of such a non-monotonic behavior is as follows. When t increases from zero, the local spin on one impurity orbital starts to feel the AFM interaction from the other impurity orbital, and thus it is screened more strongly by its surrounding environment. This results in enhanced Kondo correlation. On the other hand, when t reaches a certain value, the two local orbitals start to form delocalized molecular orbitals. Because of the AFM nature of the inter-orbital coupling, this results in an $S = 0$ ground state within the impurity, leading to the quenching of local spins. Consequently, the Kondo correlation is more and more suppressed with further increasing of t.

The situation is exactly opposite for the $\text{K}_{2'}$/Au(111) composite. Instead of the AFM t-coupling, the environment-induced SE interaction is of FM nature. Thus, when $\Delta_{\rm SE}$ increases from zero, the local spin on one impurity orbital starts to feel the FM interaction from the other impurity orbital, and thus it is screened less strongly by its surrounding environment. This results in weakened Kondo correlation. Nevertheless, when $\Delta_{\rm SE}$ reaches a certain value, the whole impurity favors an $S = 1$ ground state because of the FM coupling, resulting in a substantially enlarged spin moment within the total impurity. Consequently, the Kondo correlation is strengthened with further increasing of $\Delta_{\rm SE}$. This thus explains the non-monotonic variation of $T_{\rm K}$ versus $\Delta_{\rm SE}$ depicted in Fig. 4 of the main text.

As mentioned in Supplementary Note 6, the long-range SE interaction leads to a finite energy gap $J_{\rm eff}$ between the FM and AFM states of the $\text{K}_{2'}$/Au(111) composite. A crude estimation gives $J_{\rm eff} = E_{\rm AFM} - E_{\rm FM} \approx \gamma\frac{\Delta^2_{\rm SE}}{U+\Delta\epsilon} = 14\,\text{meV}$, but the prefactor γ does not have a simple form and thus its value is subject to a large uncertainty.

To have a more accurate assessment of $J_{\rm eff}$, we explicitly include the effective AFM t-coupling into the two-orbital AIM, and finite-tune the value of t until the resulting $S_{12} = 0$. The latter indicates that the FM and AFM spin-states become degenerate. From the HEOM calculation the zero spin correlation is reached at $t \simeq 0.027\,\text{eV}$. We thus have $J_{\rm eff} - J_t = E_{\rm AFM} - E_{\rm FM} = 0$, and so $J_{\rm eff} = J_t = \frac{4t^2}{U} \simeq 3.3\,\text{meV}$. Such a value agrees consistently with the value of 4 meV calculated by using the DFT method. Since $J_{\rm eff} < k_{\rm B}T_{\rm K}$ (the latter is about 16.5 meV), the triplet-to-singlet spin excitation is overwhelmed by the Kondo resonance and is thus invisible in the dI/dV spectra.

As shown in Supplementary Figure 19, the peak height of the PDOS of the d_{xz} orbitals

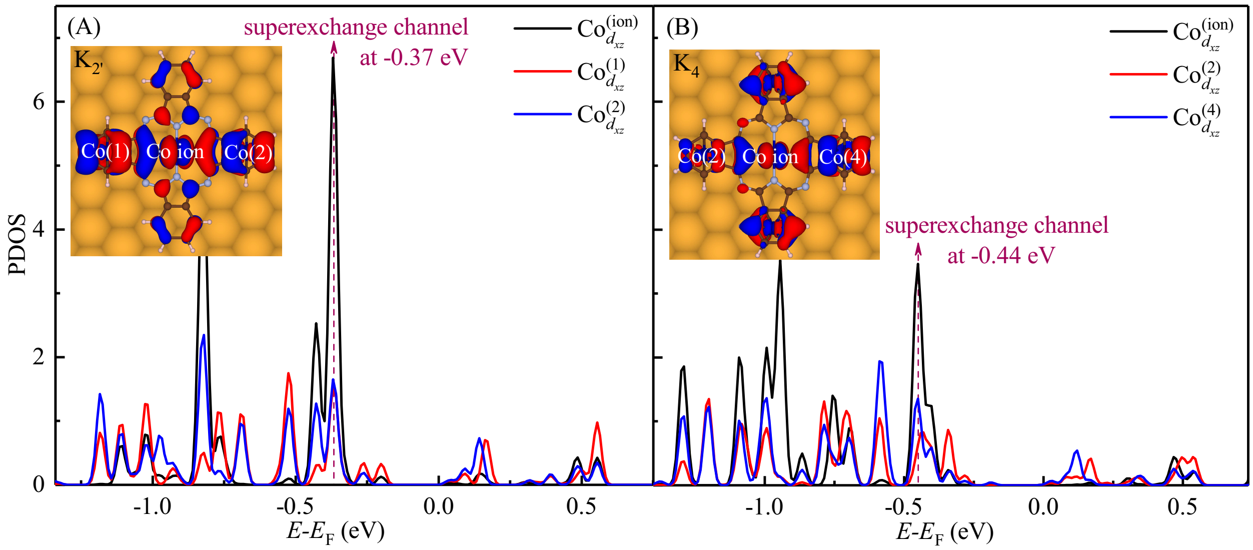

Supplementary Figure 19: PDOS of the d_{xz} orbitals of the Co adatoms and the Co ion in the (A) $K_{2'}$/Au(111) and (B) K_4/Au(111) composites, respectively. The dashed line marks the energy of the SE channel. The insets depict the delocalized Kohn-Sham orbitals (the channels) which are supposed to mediate the SE interaction.

on the captured Co adatoms and on the Co ion in a K_4/Au(111) composite (at -0.44 eV) is somewhat lower than that of a $K_{2'}$Au(111) (at -0.37 eV). This suggests the bridging channel is less effective for the two para-Co adatoms in a K_4/Au(111), and thus the SE interaction in a K_4/Au(111) is weaker than that in a $K_{2'}$/Au(111).

The delocalized Kohn-Sham orbital that mediates the SE interaction can be expressed as $\psi_{\mathrm{SE}} = \sum_j c_j \chi_j$, where j labels the basis functions including the atomic orbitals on the captured Co adatoms. Thus, $\psi_{\mathrm{SE}} = c_1 \chi_{d_{xz}}^{\mathrm{Co(1)}} + c_2 \chi_{d_{xz}}^{\mathrm{Co(2)}} + c_3 \chi_{d_{xz}}^{\mathrm{Co(ion)}} + \ldots$ for a $K_{2'}$ and $\psi'_{\mathrm{SE}} = c'_1 \chi_{d'_{xz}}^{\mathrm{Co(2)}} + c'_2 \chi_{d'_{xz}}^{\mathrm{Co(4)}} + c'_3 \chi_{d'_{xz}}^{\mathrm{Co(ion)}} + \ldots$ for a K_4. The ratio of SE interaction strength between a $K_{2'}$ and a K_4 is $\frac{\Delta_{\mathrm{SE}}(K_{2'}/\mathrm{Au(111)})}{\Delta_{\mathrm{SE}}(K_4/\mathrm{Au(111)})} \simeq \frac{\left\langle \chi_{d_{xz}}^{\mathrm{Co(1)}} | \hat{h} | \psi_{\mathrm{SE}} \right\rangle \left\langle \psi_{\mathrm{SE}} | \hat{h} | \chi_{d_{xz}}^{\mathrm{Co(2)}} \right\rangle}{\left\langle \chi_{d'_{xz}}^{\mathrm{Co(2)}} | \hat{h} | \psi'_{\mathrm{SE}} \right\rangle \left\langle \psi'_{\mathrm{SE}} | \hat{h} | \chi_{d'_{xz}}^{\mathrm{Co(4)}} \right\rangle} \simeq \frac{\left\langle \chi_{d_{xz}}^{\mathrm{Co(1)}} | \psi_{\mathrm{SE}} \right\rangle \left\langle \psi_{\mathrm{SE}} | \chi_{d_{xz}}^{\mathrm{Co(2)}} \right\rangle}{\left\langle \chi_{d'_{xz}}^{\mathrm{Co(2)}} | \psi'_{\mathrm{SE}} \right\rangle \left\langle \psi'_{\mathrm{SE}} | \chi_{d'_{xz}}^{\mathrm{Co(4)}} \right\rangle} \simeq \frac{c_1 * c_2}{c'_1 * c'_2} \simeq 8$. Since Δ_{SE} is determined to be 123 meV for a $K_{2'}$/Au(111), its value is estimated to be around 15 meV for a K_4/Au(111). Therefore, by referring again to the inset of Fig. 4 in the main text, we estimate that the SE interaction in a K_4/Au(111) could lead to a minor change in T_{K} by about 4 K.

---

[1] Nozieres, P. A Fermi-liquid description of the Kondo problem at low temperatures. *J. Low Temp. Phys.* **17**, 31–42 (1974).

[2] Kröger, J. *et al.* Surface state electron dynamics of clean and adsorbate-covered metal surfaces studied with the scanning tunnelling microscope. *Prog. Surf. Sci.* **80**, 26–48 (2005).

[3] Gruber, M., Weismann, A. & Berndt, R. The Kondo resonance line shape in scanning tunnelling spectroscopy: instrumental aspects. *J. Phys.: Condens. Matter* **30**, 424001 (2018).

[4] Otte, A. F. *et al.* The role of magnetic anisotropy in the Kondo effect. *Nat. Phys.* **4**, 847–850 (2008).

[5] Schuh, T. *et al.* Lifetimes of magnetic excitations in Fe and Co atoms and clusters on Pt(111). *J. Appl. Phys.* **107**, 09E156 (2010).

[6] Nagaoka, K., Jamneala, T., Grobis, M. & Crommie, M. Temperature dependence of a single Kondo impurity. *Phys. Rev. Lett.* **88**, 077205 (2002).

[7] Hansma, P. K. Inelastic electron tunneling. *Phys. Rep.* **30**, 145–206 (1977).

[8] Žitko, R. & Bonča, J. Multiple-impurity Anderson model for quantum dots coupled in parallel. *Phys. Rev. B* **74**, 045312 (2006).

[9] Cohen, A. J., Paula, M. S. & Yang, W. T. Challenges for density functional theory. *Chem. Rev.* **112**, 289–320 (2012).

[10] Újsághy, O., Kroha, J., Szunyogh, L. & Zawadowski, A. Theory of the Fano resonance in the STM tunneling density of states due to a single Kondo impurity. *Phys. Rev. Lett.* **85**, 2557–2560 (2000).

[11] Hewson, A. C. *The Kondo problem to heavy Fermions*, vol. 2 (Cambridge Univ. Press, Cambridge, 1997).

[12] Wang, Y., Zheng, X., Li, B. & Yang, J. L. Understanding the Kondo resonance in the d-CoPc/Au (111) adsorption system. *J. Chem. Phys.* **141**, 084713 (2014).

[13] Zhao, A. D. *et al.* Controlling the Kondo effect of an adsorbed magnetic ion through its chemical bonding. *Science* **309**, 1542–1544 (2005).

[14] Costi, T. A., Hewson, A. C. & Zlatic, V. Transport coefficients of the Anderson model via the numerical renormalization group. *J. Phys.: Condens. Matter* **6**, 2519–2558 (1994).

[15] Goldhaber-Gordon, D. *et al.* From the Kondo regime to the mixed-valence regime in a single-electron transistor. *Phys. Rev. Lett.* **81**, 5225–5228 (1998).

[16] Mugarza, A. *et al.* Spin coupling and relaxation inside molecule-metal contacts. *Nat. Commun.* **2**, 490 (2011).

[17] Li, Z. *et al.* Kondo-peak splitting and resonance enhancement caused by interdot tunneling in coupled double quantum dots. *Phys. Rev. B* **98**, 115133 (2018).

[18] Lopez, R., Aguado, R. & Platero, G. Nonequilibrium transport through double quantum dots: Kondo effect versus antiferromagnetic coupling. *Phys. Rev. Lett.* **89**, 136802 (2002).
