# Supplementary material for: Molecular molds for regularizing Kondo states at atom/metal interfaces
Source: Nat Commun. 2020 May 22;11:2566. doi: 10.1038/s41467-020-16402-6 (PMC7244723; doi:10.1038/s41467-020-16402-6)
Supplement: Supplementary file 3 — Description of Additional Supplementary Files [file 41467_2020_16402_MOESM3_ESM.pdf]

Description of Additional Supplementary Files

File name: Supplementary Movie 1

Description: A movie showing the STM manipulation of a CoPc molecular mold to capture dispersed Co adatoms on the Au(111) surface
