# Supplementary figures and images for: Molecular molds for regularizing Kondo states at atom/metal interfaces
Source: Nat Commun. 2020 May 22;11:2566. doi: 10.1038/s41467-020-16402-6 (PMC7244723; doi:10.1038/s41467-020-16402-6)

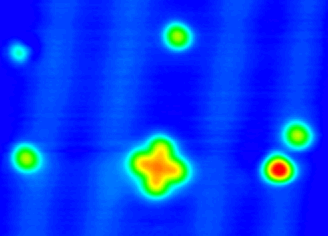

Supplement: Supplementary file 4 — Supplementary Movie 1 [file 41467_2020_16402_MOESM4_ESM.gif]
